# The Prevalence and Trends of Contrast-Induced Nephropathy Among Hospitalized Patients Undergoing Cardiac Resynchronization Therapy

**DOI:** 10.7759/cureus.97332

**Published:** 2025-11-20

**Authors:** Charles O Poluyi, Omar A Oudit, Jamal C Perry, Oluwasegun M Akinti, Ajibola Adedayo, Emmanuel Ukenenye, Victor Onowori

**Affiliations:** 1 Internal Medicine, Brookdale University Hospital Medical Center, Brooklyn, USA; 2 Medicine, Brookdale University Hospital Medical Center, Brooklyn, USA; 3 Interventional Cardiology, Interfaith Medical Center, Brooklyn, USA; 4 General Practice, Adeolu Hospital, Lagos, NGA

**Keywords:** acute kidney injury, cardiac resynchronization therapy, chronic kidney disease, contrast-induced nephropathy, heart failure, hospital readmission

## Abstract

Introduction: Contrast-induced nephropathy (CIN) is a significant complication following the use of iodinated contrast agents during cardiac procedures. Cardiac resynchronization therapy (CRT), increasingly used in heart failure management, requires contrast administration, putting patients at risk for CIN. However, the prevalence and outcomes of CIN in CRT recipients remain underexplored.

Methods: We conducted a retrospective cohort study using the Nationwide Readmissions Database (NRD) from 2016 to 2020. Adult patients undergoing CRT or CRT-D implantation were identified using International Classification of Diseases, 10th edition (ICD-10) codes. The primary outcome was the annual prevalence of CIN. Secondary outcomes included 30-day hospital readmission, mortality, metabolic acidosis, hyperkalemia, and continuous renal replacement therapy (CRRT). Multivariable logistic regression assessed temporal trends, adjusting for patient demographics and comorbidities.

Results: Among 42,545 patients undergoing CRT, CIN prevalence increased from 20.61% in 2016 to 26.40% in 2020 (p < 0.001). CIN was associated with significantly higher 30-day readmission rates, longer hospital stays (mean length of stay (LOS): 7.64 vs. 4.36 days in non-elective cases), and higher hospitalization costs. The incidence of metabolic acidosis rose from 13.73% to 18.51% (p < 0.001), and CRRT use increased from 3.30% to 5.11% (p = 0.03). While overall mortality was higher among CIN patients (2.84% vs. 0.53%), no significant trend in mortality was observed over time (p = 0.352).

Conclusion: CIN is an increasingly prevalent complication in patients undergoing CRT and is associated with worse clinical outcomes and higher healthcare utilization. These findings underscore the need for implementing standardized nephroprotective strategies, including contrast minimization protocols and early risk stratification, to mitigate renal complications in this high-risk population.

## Introduction

Heart failure (HF) is a leading cause of global morbidity and mortality, with management often requiring a combination of pharmacotherapy and device-based interventions [1]. Among these, cardiac resynchronization therapy (CRT) has emerged as a vital strategy for patients with advanced HF and a prolonged QRS duration (≥130 ms) despite optimal pharmacotherapy [2]. CRT enhances mechanical synchrony by pacing both the left ventricular (LV) free wall and septum, improving LV ejection fraction (LVEF), survival rates, six-minute walk distance, and QRS duration [3]. As HF prevalence rises and CRT indications expand, the procedure is performed more frequently worldwide [4].

A thorough evaluation of coronary sinus (CS) anatomy using coronary venous angiography is required to optimize LV lead placement. This procedure necessitates contrast administration, which carries the risk of contrast-induced nephropathy (CIN) [5]. The most frequent cause of failure in CRT device placement is the inability to cannulate the CS ostium [6] successfully. However, using contrast material in this patient population introduces the risk of CIN [7]. The development of CIN following CRT has a significant negative impact on morbidity and long-term prognosis [2].

The prevalence and metrics of CIN among individuals undergoing CRT implantation remain relatively understudied within the existing literature. This study aimed to evaluate the prevalence, clinical outcomes, and economic impact of CIN among CRT recipients using U.S. Nationwide Readmissions Database (NRD) data from 2016 to 2020, and to highlight the growing burden of CIN and the need for standardized nephroprotective strategies to improve patient care.

## Materials and methods

Study data

The National Inpatient Sample (NIS) is part of the Healthcare Cost and Utilization Project (HCUP) and is maintained by the Agency for Healthcare Research and Quality (AHRQ) [8]. The NIS contains information on all inpatient stays (not individual patients) in 48 states plus the District of Columbia, representing approximately 98% of the U.S. population, excluding rehabilitation and long-term acute care hospitals [8]. Unweighted, it contains data from more than seven million hospital stays each year, and weighted, it estimates more than 35 million hospitalizations nationally [8]. All discharge diagnoses and procedures are identified using the International Classification of Diseases, 10th edition (ICD-10) codes [6]. The AHRQ made these data available to the principal author via the HCUP. The NRD, for the years 2016 to 2020, has been the largest publicly available all-payer inpatient healthcare readmission database. The NRD includes all-payer hospital discharges from up to 30 states. It comprises over 60% of all U.S. hospitalizations in community, public, academic, general acute care, and specialty hospitals.

Study population

We included all patients with ICD-10-CM and ICD-10-PCS procedure codes for CRT and CRT-D placement, excluding those younger than 18 years. CIN was identified using the ICD-10-CM code N14.1 (nephropathy due to drugs or biological substances, including contrast media) when listed as a secondary diagnosis during the index CRT admission. We also included N17.x codes for acute kidney injury (AKI) to reflect standard coding practice for post-contrast AKI in national datasets. Prior validation studies show that ICD-10 codes for AKI have good specificity and positive predictive value, though they may miss milder cases. To improve accuracy, we also performed a sensitivity analysis limited to the N14.1 code to focus on cases most likely representing true CIN. For the NRD, we excluded elective admissions and index hospitalizations discharged in December to ensure complete 30-day readmission follow-up within the same calendar year.

Outcome measures

Trends in Outcomes for 30-Day Hospital Readmission

Trends in outcomes for 30-day hospital readmission were analyzed using NRD data, where each patient was assigned a unique identification number to track admissions within the state for each patient from 2016 to 2020. A readmission was defined as any non-traumatic admission for any principal diagnosis within 30 days of the index admission. Only the first readmission was counted if patients had multiple readmissions within 30 days of discharge. Patients who died during index admission were excluded from the denominator.

Temporal Trends in CIN Rates in CRT/CRT-D

We initially examined the temporal trends of CIN rates in patients undergoing CRT/CRT-D in the overall cohort. The primary outcomes of interest included mortality, metabolic acidosis, hyperkalemia, and continuous renal replacement therapy (CRRT).

Statistical analysis

We used Student's t-test for continuous variables to evaluate temporal changes in baseline characteristics and the χ² test for categorical variables. Multiple logistic and linear regression analyses were conducted to assess yearly trends, with the year of admission as the independent variable and the Cochrane-Armitage test for trends in categorical data. The primary outcomes were adjusted for age, sex, and chronic kidney disease (CKD).

We constructed unadjusted and adjusted multivariate regression models for the overall cohort to assess whether outcomes have improved over time. The calendar year was entered as a continuous variable to obtain unadjusted and adjusted odds ratios (ORs) per year for the overall temporal trend in outcomes and as a categorical variable with 2016 as the reference year to evaluate year-to-year variability. We compared trends in readmission rates between subgroups using a subgroup-year interaction term in multivariate regression. Results were reported as adjusted ORs with 95% confidence intervals (CIs) and beta coefficients (Coef.) with 95% CIs. A two-sided p-value of <0.05 was considered statistically significant. All analyses were conducted using STATA, version 18 (StataCorp, College Station, TX, U.S.).

## Results

Baseline characteristics

The study included 42,545 patients with a mean age of 72 years. The cohort consisted of 71.25% male and 28.75% female patients. The racial composition included 72.20% White, 14.85% Black, 8.08% Hispanic, and 1.80% Asian individuals. Hospital regions were distributed as follows: South (41.90%), Midwest (23.32%), Northeast (17.81%), and West (16.97%). Most patients received treatment in large hospitals (63.05%).

Income distribution showed that 28.07% of patients were in the lowest income bracket, 27.26% in the median range, 24.61% in the 75th percentile, and 20.07% in the highest income bracket. Insurance coverage was predominantly through Medicare (77.02%), followed by private insurance (14.35%), Medicaid (6.88%), and uninsured patients (1.76%).

The average length of stay (LOS) for procedures performed within 24 hours (elective) was 2.51 days (95% CI 2.43-2.59) for patients without CIN. In contrast, the mean LOS was significantly longer for patients with CIN at 5.84 days (95% CI 5.38-6.30). Similarly, for procedures performed after 24 hours (non-elective), the LOS was 4.36 days (95% CI 4.34-4.37) in patients without CIN, while it increased to 7.64 days (95% CI 7.60-7.68) for those with CIN.

The mean total cost for elective procedures without CIN was $37,049.02 (95% CI $36,401.53-$37,696.50), whereas the price was substantially higher at $44,383.71 (95% CI $42,761.88-$46,005.53) for patients with CIN. For non-elective procedures, the mean cost for patients without CIN was $52,062.36 (95% CI $51,225.25-$52,899.46), while for those with CIN, the price significantly increased to $66,463.47 (95% CI $64,926.90-$68,000.03).

In the CIN group, 44.18% of patients were over 75 years old, 48.54% had diabetes mellitus, 20.97% were obese, 37.17% used tobacco, 2.89% had intra-aortic balloon pumps (IABP), 62.14% had CKD, 18.54% had anemia, 17.38% had hypotension, and 6.77% had hypertension. Comparatively, in the non-CIN group, 40.00% were over 75 years old, 41.38% had diabetes mellitus, 18.37% were obese, 41.09% used tobacco, 0.74% had IABP, 39.19% had CKD, 9.09% had anemia, 11.50% had hypotension, and 19.10% had hypertension.

The Charlson Comorbidity Index (CCI) showed 81.98% of CIN patients had three or more comorbidities, compared to 61.25% of non-CIN patients. Baseline clinical and demographic characteristics of patients stratified by the presence or absence of CIN are summarized in Table 1.

**Table 1:** Overall patient characteristics of the study cohort stratified by the presence or absence of contrast-induced nephropathy (CIN) Continuous variables were compared using Student’s t-test, and categorical variables were compared using the chi-squared (χ²) test. A two-sided p-value of <0.05 was considered statistically significant. Corresponding test statistics (t or χ² values) are reported alongside each variable. CI: confidence interval; IABP: intra-aortic balloon pump

Variables	CIN	Non-CIN	Statistical test	p-value
Age (mean years)	72	72	Student's t-test (t = 0.0)	1.00
Gender: male (%)	71.25% (n = 7,578)	71.25% (n = 22,735)	Chi-squared test (χ² = 0.0)	≥0.05
Race: White (%)	72.20% (n = 7,679)	72.20% (n = 23,038)	Chi-squared test (χ² = 0.0)	≥0.05
Length of stay ≤ 1 day (mean, 95% CI)	5.84 (5.38-6.30)	2.51 (2.43-2.59)	Student's t-test (t = 13.98)	<0.001
Length of stay > 1 day (mean, 95% CI)	7.64 (7.60–7.68)	4.36 (4.34–4.37)	Student's t-test (t = 150.49)	<0.001
Total cost ≤ 1 day (mean, 95% CI)	$44,383.71 (42,761.88–46,005.53)	$37,049.02 (36,401.53–37,696.50)	Student's t-test (t = 8.23)	<0.001
Total cost > 1 day (mean, 95% CI)	$66,463.47 (64,926.90–68,000.03)	$52,062.36 (51,225.25–52,899.46)	Student's t-test (t = 16.13)	<0.001
>75 years old (%)	44.18% (n = 4,699)	40.00% (n = 12,764)	Chi-squared test (χ² = 57.57)	<0.001
Diabetes mellitus (%)	48.54% (n = 5,163)	41.38% (n = 13,204)	Chi-squared test (χ² = 166.81)	<0.001
Obesity (%)	20.97% (n = 2,230)	18.37% (n = 5,862)	Chi-squared test (χ² = 34.89)	<0.001
Tobacco use (%)	37.17% (n = 3,953)	41.09% (n = 13,111)	Chi-squared test (χ² = 51.09)	<0.001
IABP use (%)	2.89% (n = 307)	0.74% (n = 236)	Chi-squared test (χ² = 291.78)	<0.001
Chronic kidney disease (CKD) (%)	62.14% (n = 6,609)	39.19% (n = 12,505)	Chi-squared test (χ² = 1,697.87)	<0.001
Anemia (%)	18.54% (n = 1,972)	9.09% (n = 2,901)	Chi-squared test (χ² = 702.31)	<0.001
Hypotension (%)	17.38% (n = 1,849)	11.50% (n = 3,670)	Chi-squared test (χ² = 244.54)	<0.001
Hypertension (%)	6.77% (n = 720)	19.10% (n = 6,095)	Chi-squared test (χ² = 901.76)	<0.001

CIN trends

**Figure 1:**
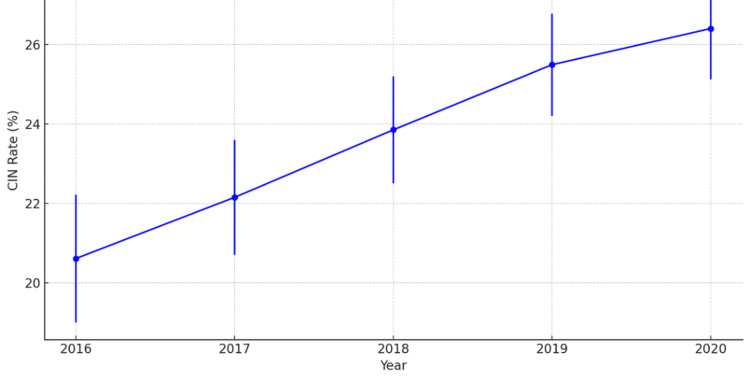
Trend in adjusted CIN rates (2016 to 2020) CIN: contrast-induced nephropathy

The adjusted CIN rate was 20.61% in 2016. This rate increased to 22.15% in 2017 (OR 1.10; 95% CI 1.00-1.21; p = 0.043). Over subsequent years, the rates continued to rise, reaching 23.85% in 2018 (OR 1.22; 95% CI 1.11-1.35; p < 0.001), escalating to 25.49% in 2019 (OR 1.35; 95% CI 1.23-1.48; p < 0.001), and peaking at 26.40% in 2020 (OR 1.42; 95% CI 1.28-1.56; p < 0.001) (trend p < 0.001). Tables 2, 3 present the adjusted and unadjusted rates of CIN, respectively, along with the corresponding ORs, highlighting year-to-year differences. Figure 1 demonstrates a steady upward trend in adjusted CIN rates from 2016 to 2020. Error bars in Figure 1 represent 95% CIs, indicating the variability around each estimate.

**Table 2:** Annual trends in adjusted contrast-induced nephropathy (CIN) rates from 2016 to 2020 with corresponding odds ratios (ORs) and 95% confidence intervals (CIs) Adjusted ORs were derived from multivariable logistic regression models controlling for age, sex, and comorbidities. A two-sided p-value of <0.05 was considered statistically significant.

Year	Adjusted CIN rate	OR (95% CI)	p-value
2016	20.61%	Reference	
2017	22.15%	1.10 (1.00-1.21)	0.043
2018	23.85%	1.22 (1.11-1.35)	<0.001
2019	25.49%	1.35 (1.23-1.48)	<0.001
2020	26.40%	1.42 (1.28-1.56)	<0.001

**Table 3:** Annual trends in unadjusted contrast-induced nephropathy (CIN) rates from 2016 to 2020 with corresponding odds ratios (ORs) and 95% confidence intervals (CIs) Unadjusted ORs were calculated using univariable logistic regression models. A two-sided p-value of <0.05 was considered statistically significant.

Year	Unadjusted CIN rate	OR (95% CI)	p-value
2016	27.65%	Reference	
2017	30.08%	1.13 (1.03-1.23)	0.011
2018	32.37%	1.25 (1.14-1.37)	<0.001
2019	34.61%	1.39 (1.27-1.51)	<0.001
2020	35.33%	1.43 (1.30-1.57)	<0.001

Readmission rates

**Figure 2:**
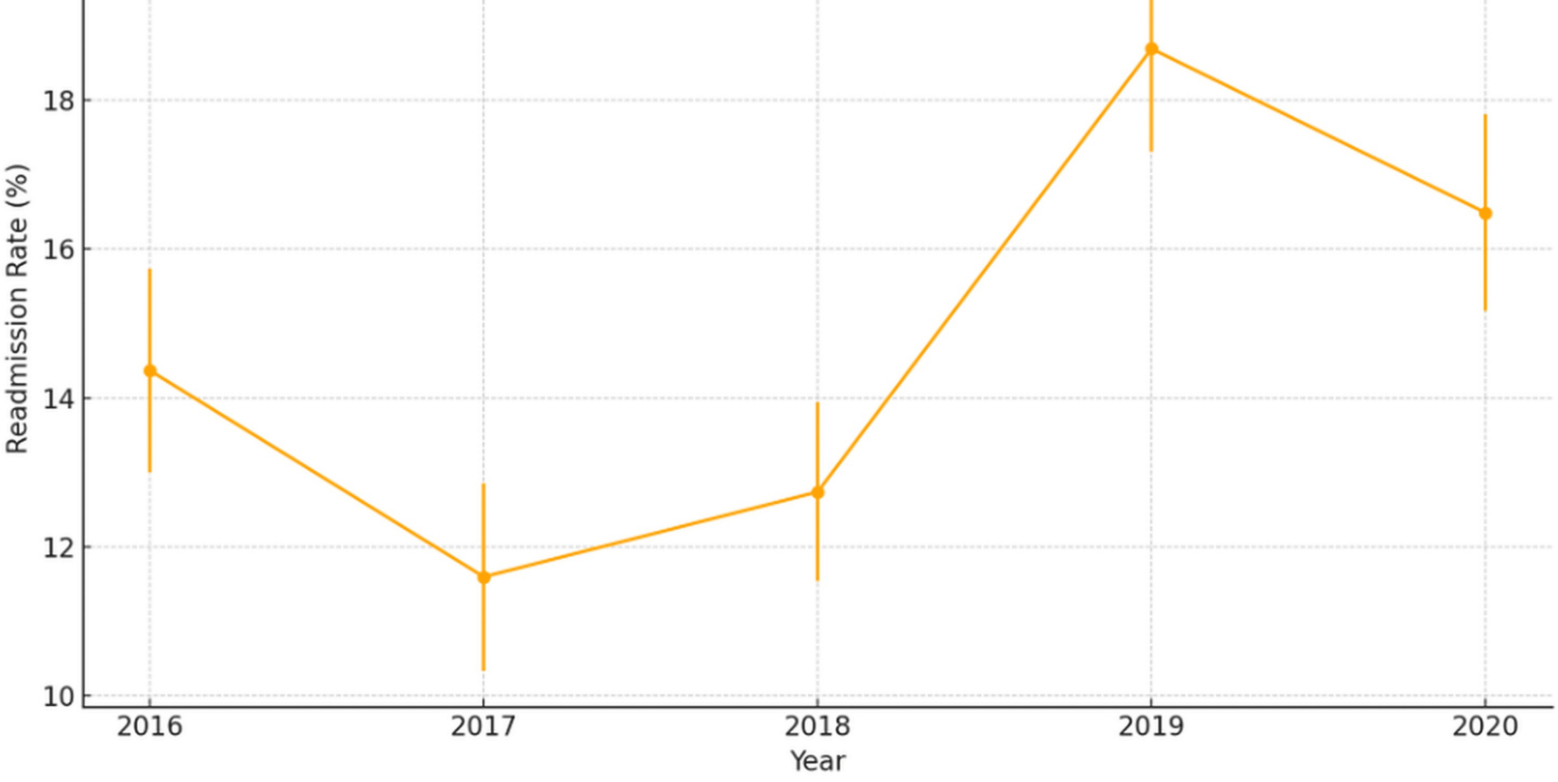
Trend in adjusted 30-day readmission rates (2016–2020)

The adjusted 30-day readmission rate was 14.37% in 2016. In 2017, this rate decreased to 11.60% (OR 0.77; 95% CI 0.67-0.90; p = 0.001). Over the subsequent years, the rates fluctuated, increasing to 12.74% in 2018 (OR 0.85; 95% CI 0.74-0.98; p = 0.028), further rising to 18.69% in 2019 (OR 1.17; 95% CI 1.03-1.33; p = 0.017), and slightly decreasing to 16.49% in 2020 (OR 1.12; 95% CI 0.98-1.27; p = 0.09) (trend p < 0.001). Tables 4, 5 present the adjusted and unadjusted rates, respectively, along with their corresponding ORs and statistical significance, highlighting key year-to-year differences. Figure 2 illustrates the adjusted readmission rates, demonstrating a notable increase between 2018 and 2019, followed by a modest decline in 2020. Error bars in Figure 2 represent 95% CIs, indicating the precision of the estimates.

**Table 4:** Annual trends in adjusted 30-day readmission rates from 2016 to 2020 with corresponding odds ratios (ORs) and 95% confidence intervals (CIs) Adjusted ORs were derived from multivariable logistic regression models controlling for age, sex, and comorbidities. A two-sided p-value of <0.05 was considered statistically significant.

Year	Adjusted readmission rate	OR (95% CI)	p-value
2016	14.37%	Reference	-
2017	11.60%	0.77 (0.67-0.90)	0.001
2018	12.74%	0.85 (0.74-0.98)	0.028
2019	18.69%	1.17 (1.03-1.33)	0.017
2020	16.49%	1.12 (0.98-1.27)	0.09

**Table 5:** Annual trends in unadjusted 30-day readmission rates from 2016 to 2020 with corresponding odds ratios (ORs) and 95% confidence intervals (CIs) Unadjusted ORs were calculated using univariable logistic regression models. A two-sided p-value of <0.05 was considered statistically significant.

Year	Unadjusted readmission rate	OR (95% CI)	p-value
2016	18.84%	Reference	-
2017	15.19%	0.78 (0.67-0.90)	0.001
2018	16.43%	0.85 (0.74-0.98)	0.028
2019	20.10%	1.17 (1.03-1.32)	0.017
2020	20.23%	1.11 (0.98-1.26)	0.107

Mortality rates

**Figure 3:**
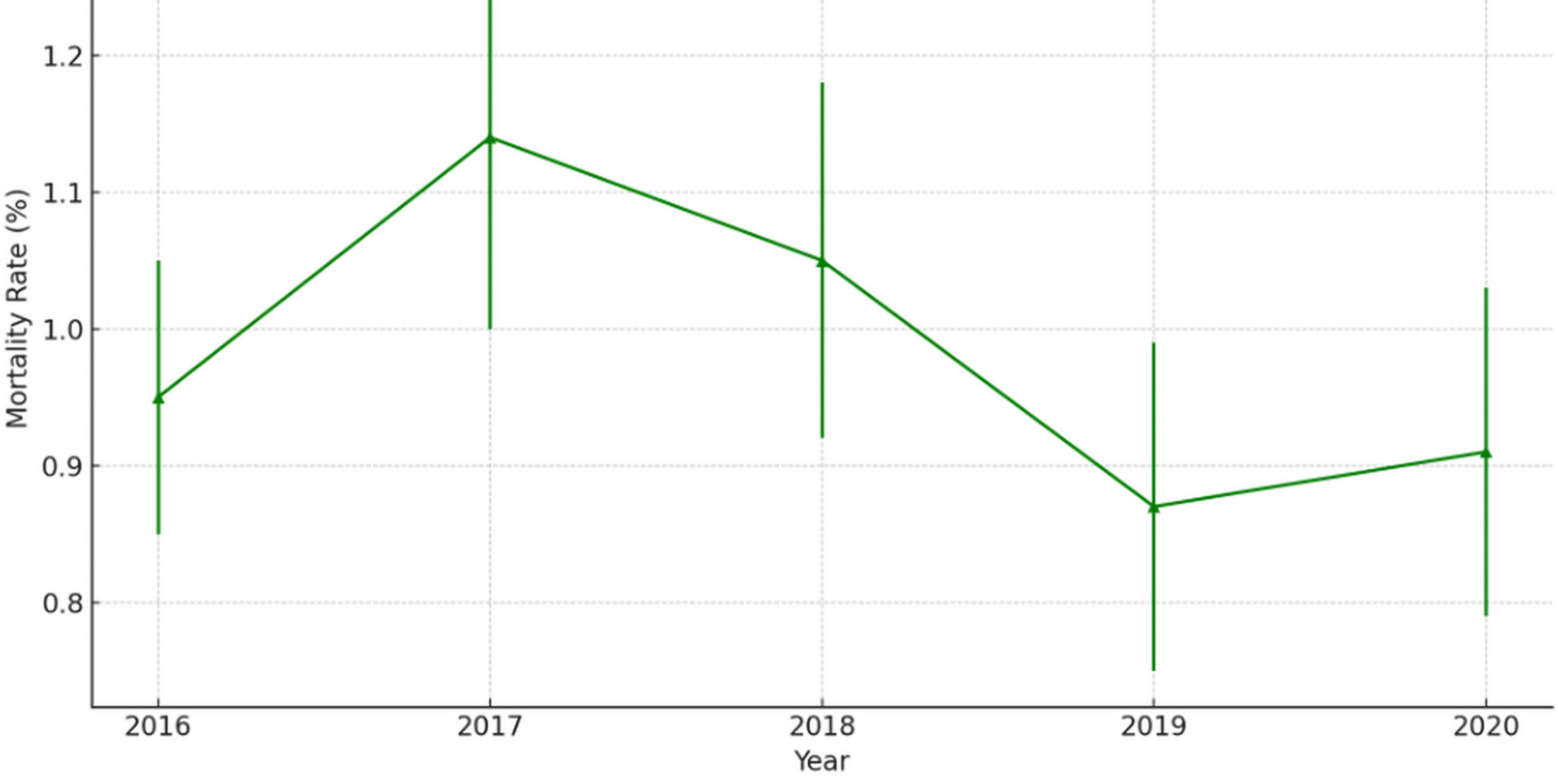
Trend in adjusted in-hospital mortality rates (2016–2020)

The overall mortality rate was 0.53% in patients without CIN compared to 2.84% in those with CIN. The adjusted mortality rate was 0.95% in 2016. This rate increased to 1.14% in 2017 (OR 1.20; 95% CI 0.85-1.69; p = 0.293). Over subsequent years, the rates fluctuated, decreasing to 1.05% in 2018 (OR 1.11; 95% CI 0.79-1.54; p = 0.558), further dropping to 0.87% in 2019 (OR 0.91; 95% CI 0.65-1.29; p = 0.608), and then slightly increasing to 0.91% in 2020 (OR 0.96; 95% CI 0.66-1.38; p = 0.807) (trend p = 0.352). Tables 6, 7 present the unadjusted and adjusted in-hospital mortality rates, respectively, along with their corresponding ORs, highlighting year-to-year differences. Figure 3 depicts the trend in adjusted in-hospital mortality rates from 2016 to 2020. Error bars in Figure 3 represent 95% CIs, reflecting the variability around the point estimates.

**Table 6:** Annual trends in unadjusted in-hospital mortality rates from 2016 to 2020 with corresponding odds ratios (ORs) and 95% confidence intervals (CIs) Unadjusted ORs were calculated using univariable logistic regression models. A two-sided p-value of <0.05 was considered statistically significant.

Year	Unadjusted mortality rate	OR (95% CI)	p-value
2016	3.03%	Reference	-
2017	3.18%	1.05 (0.69-1.62)	0.817
2018	3.15%	1.04 (0.70-1.56)	0.846
2019	2.72%	0.90 (0.60-1.34)	0.593
2020	2.10%	0.69 (0.44-1.08)	0.103

**Table 7:** Annual trends in adjusted in-hospital mortality rates from 2016 to 2020 with corresponding odds ratios (ORs) and 95% confidence intervals (CIs) Adjusted ORs were derived from multivariable logistic regression models controlling for age, sex, and comorbidities. A two-sided p-value of <0.05 was considered statistically significant.

Year	Adjusted mortality rate	OR (95% CI)	p-value
2016	0.95%	Reference	-
2017	1.14%	1.20 (0.85-1.69)	0.293
2018	1.05%	1.11 (0.79-1.54)	0.558
2019	0.87%	0.91 (0.65-1.29)	0.608
2020	0.91%	0.96 (0.66-1.38)	0.807

Metabolic acidosis trends

**Figure 4:**
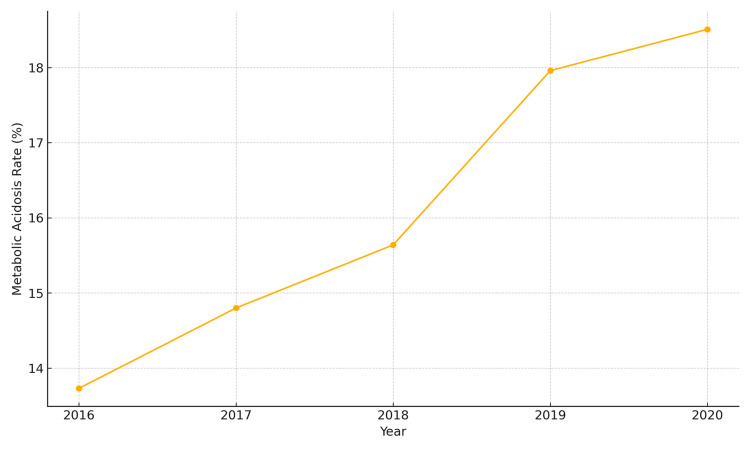
Trend in metabolic acidosis from 2016 to 2020

The rate of metabolic acidosis was 13.73% in 2016. This increased to 14.80% in 2017 (OR 1.09; 95% CI 0.88-1.35; p = 0.417). The rate continued to rise-reaching 15.64% in 2018 (OR 1.16; 95% CI 0.95-1.42; p = 0.133), escalating to 17.96% in 2019 (OR 1.38; 95% CI 1.13-1.67; p = 0.001), and peaking at 18.51% in 2020 (OR 1.49; 95% CI 1.17-1.74; p < 0.001) (trend p < 0.001). Figure 4 illustrates the rising trend in the incidence of metabolic acidosis from 2016 to 2020, increasing from 13.7% to 18.5% over the study period.

Hyperkalemia and CRRT utilization

The rate of hyperkalemia fluctuated, with a slight decline from 14.74% in 2016 to 14.04% in 2017 (OR 0.94; 95% CI 0.76-1.17; p = 0.601). It increased to 16.07% in 2019 (OR 1.11; 95% CI 0.91-1.35; p = 0.315) before declining slightly to 14.03% in 2020 (OR 0.91; 95% CI 0.77-1.15; p = 0.574) (trend p = 0.79).

**Figure 5:**
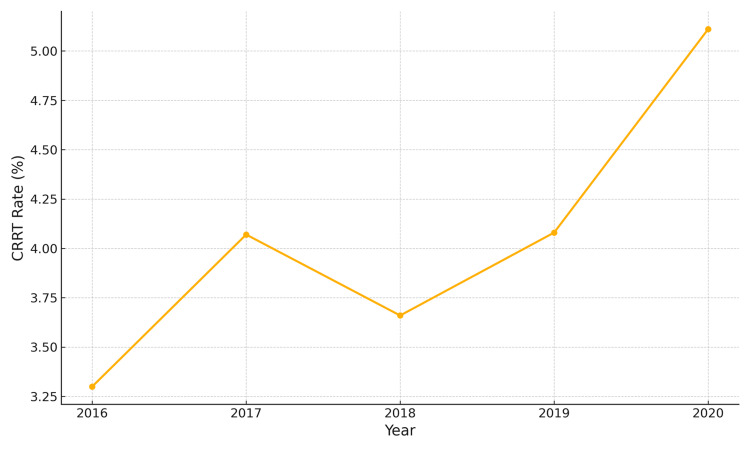
Trend in CRRT utilization from 2016 to 2020 CRRT: continuous renal replacement therapy

The rate of CRRT was 3.30% in 2016, increasing to 5.11% in 2020 (OR 1.58; 95% CI 1.08-2.31; p = 0.018) (trend p = 0.03). Figure 5 shows a progressive increase in the use of CRRT over the five-year period, rising from 3.3% in 2016 to 5.1% in 2020.

## Discussion

The main finding of this study is the rising prevalence of CIN in patients undergoing CRT, increasing from 20.61% in 2016 to 26.40% in 2020 (p < 0.001). This trend suggests that CIN remains a significant and growing concern among CRT recipients. The increase is likely due to greater CRT utilization, higher-risk patient profiles, evolving procedural techniques requiring more contrast volume, and improved detection/documentation of CIN cases. Given CIN's strong association with adverse outcomes, including increased hospital readmission rates, prolonged LOS, and higher healthcare costs, these findings emphasize the urgent need for improved preventive strategies to mitigate contrast-related kidney injury in CRT patients.

CIN was also associated with higher 30-day readmission rates, extended hospital stays, and increased hospitalization costs, reinforcing its substantial impact on healthcare utilization. The 30-day readmission rate fluctuated over time, initially decreasing to 11.60% in 2017, then rising to 18.69% in 2019, and finally stabilizing at 16.49% in 2020 (p < 0.001). Patients who developed CIN had significantly longer hospital stays (5.84 vs. 2.51 days in elective cases; 7.64 vs. 4.36 days in non-elective cases) and higher hospitalization costs ($66,463.47 for non-elective CIN patients vs. $52,062.36 for non-CIN patients). These findings highlight the economic burden associated with CIN and reinforce the need for proactive interventions, such as contrast minimization protocols and nephroprotective strategies, to reduce these financial implications [7,9].

The rising incidence of metabolic acidosis and the need for CRRT in CIN patients further underscores the clinical importance of this condition. The prevalence of metabolic acidosis increased from 13.73% in 2016 to 18.51% in 2020 (p < 0.001), while CRRT utilization nearly doubled (3.30% to 5.11%, p = 0.03), suggesting that CIN is not merely a transient renal dysfunction but can lead to severe metabolic and hemodynamic instability requiring advanced renal support [10]. Previous studies have shown that CIN exacerbates electrolyte imbalances and acid-base disturbances and increases the risk of AKI, particularly in high-risk patients such as those with CKD, diabetes, or hemodynamic instability [11]. Given these risks, early identification and management of CIN are crucial to preventing progression to severe renal failure and associated complications.

Despite the clear impact of CIN on morbidity, healthcare costs, and hospital readmission, this study did not identify a significant trend in overall mortality (p = 0.352). While CIN patients had a higher overall mortality rate (2.84%) compared to non-CIN patients (0.53%), the lack of a consistent increase in mortality over time may suggest advancements in CRT technology, HF management, and renal protective strategies that have improved patient outcomes despite CIN development [12]. This contrasts with earlier studies in percutaneous coronary intervention (PCI) populations, where CIN was found to be an independent predictor of mortality [6]. The findings may reflect variations in procedural complexity, patient characteristics, and advancements in post-procedural care among CRT recipients.

The increasing prevalence of CIN in CRT patients observed in this study is consistent with prior research demonstrating renal risks associated with contrast-based procedures in cardiovascular medicine. The observed increase in CIN from 20.61% in 2016 to 26.40% in 2020 (p < 0.001) aligns with earlier reports that identified CIN as a frequently overlooked yet clinically significant complication in CRT recipients [12]. A subanalysis of the TRUST CRT trial reported CIN rates between 10% and 25%, comparable to our findings. However, our study demonstrates a continuing upward trajectory, possibly due to more frequent CRT utilization in high-risk patients and improvements in CIN recognition [2].

Our findings further support the link between CIN and increased hospital readmission rates, prolonged hospital stays, and greater healthcare expenditures [8]. Previous studies on contrast-induced kidney injury in PCI populations have shown that CIN is a significant determinant of extended hospitalization, higher morbidity, and increased readmissions due to worsening renal function and associated complications [6]. Similarly, research on cardiovascular procedures has documented a two- to threefold increase in hospital stays and readmission rates in CIN patients, findings that align with the significant economic impact observed in this study [13].

Beyond economic considerations, our study highlights the association between CIN and worsening metabolic and renal outcomes, including an increased need for CRRT [11]. Previous research has demonstrated that CIN is a leading cause of AKI requiring dialysis, particularly in patients with pre-existing CKD or diabetes, who are at heightened risk for renal deterioration following contrast exposure [12].

Given the increasing prevalence of CIN and its association with worse outcomes, there is an urgent need to incorporate nephroprotective strategies into CRT protocols, especially for patients with pre-existing CKD or diabetes [10]. Strategies such as contrast minimization, use of low- or iso-osmolar contrast agents, and pre-procedural hydration optimization should be prioritized to reduce CIN risk. Hospitals should implement CIN prevention pathways and risk-stratification tools to improve patient outcomes and reduce healthcare expenditures [14].

Post-procedural monitoring should be strengthened to detect early signs of renal deterioration. Routine serum creatinine and electrolyte monitoring should be mandated within 48-72 hours post-CRT implantation, with early nephrology consultation for high-risk patients.

Limitations

This study has several limitations. Because it is retrospective and relies on administrative ICD-10 data, there is a possibility of misclassifying cases of CIN. To reduce this risk, we used the ICD-10-CM code N14.1 (nephropathy due to drugs or biological substances, including contrast media), which has been validated in prior national database studies. Earlier work shows that ICD-10 codes for AKI have good specificity and positive predictive value but may under-detect milder cases. In addition, the NRD does not include details such as contrast volume, baseline estimated glomerular filtration rate (eGFR), or exposure to nephrotoxic medications, all of which could affect CIN risk and limit reproducibility across settings. We acknowledge these constraints to maintain transparency and strengthen the methodological rigor of our findings.

## Conclusions

This study suggests a rising burden of CIN among patients undergoing CRT between 2016 and 2020. CIN was associated with higher 30-day readmission rates, longer hospital stays, greater use of renal replacement therapy, and increased healthcare costs. These findings highlight the growing clinical and economic impact of CIN in this high-risk group and stress the importance of using consistent nephroprotective strategies such as contrast minimization, risk assessment, and early kidney monitoring to reduce complications. Future prospective studies are needed to confirm these trends, explore long-term kidney and heart outcomes, and evaluate safer, contrast-sparing techniques for CRT patients with underlying risk factors like CKD and diabetes.

## References

[1] Benjamin EJ, Virani SS, Callaway CW (2018). Heart Disease and Stroke Statistics-2018 update: a report from the American Heart Association. Circulation.

[2] Ponikowski P, Voors AA, Anker SD (2016). 2016 ESC guidelines for the diagnosis and treatment of acute and chronic heart failure: the task force for the diagnosis and treatment of acute and chronic heart failure of the European Society of Cardiology (ESC)developed with the special contribution of the Heart Failure Association (HFA) of the ESC. Eur Heart J.

[3] McAlister FA, Ezekowitz J, Hooton N (2007). Cardiac resynchronization therapy for patients with left ventricular systolic dysfunction: a systematic review. JAMA.

[4] Boriani G, Diemberger I (2017). Cardiac resynchronization therapy in the real world: need to focus on implant rates, patient selection, co-morbidities, type of devices, and complications. Eur Heart J.

[5] Singh JP, Klein HU, Huang DT (2011). Left ventricular lead position and clinical outcome in the multicenter automatic defibrillator implantation trial-cardiac resynchronization therapy (MADIT-CRT) trial. Circulation.

[6] Marenzi G, Lauri G, Assanelli E (2004). Contrast-induced nephropathy in patients undergoing primary angioplasty for acute myocardial infarction. J Am Coll Cardiol.

[7] Cowburn PJ, Patel H, Pipes RR, Parker JD (2005). Contrast nephropathy post cardiac resynchronization therapy: an under-recognized complication with important morbidity. Eur J Heart Fail.

[8] He H, Chen XR, Chen YQ, Niu TS, Liao YM (2019). Prevalence and predictors of contrast-induced nephropathy (CIN) in patients with ST-segment elevation myocardial infarction (STEMI) undergoing percutaneous coronary intervention (PCI): a meta-analysis. J Interv Cardiol.

[9] Kowalczyk J, Lenarczyk R, Kowalski O (2014). Contrast-induced acute kidney injury in patients undergoing cardiac resynchronization therapy-incidence and prognostic importance. Sub-analysis of data from randomized TRUST CRT trial. J Interv Card Electrophysiol.

[10] McCullough PA, Wolyn R, Rocher LL, Levin RN, O'Neill WW (1997). Acute renal failure after coronary intervention: incidence, risk factors, and relationship to mortality. Am J Med.

[11] Bradley DJ (2003). Combining resynchronization and defibrillation therapies for heart failure. JAMA.

[12] Phillips A, Shaper AG, Whincup PH (1989). Association between serum albumin and mortality from cardiovascular disease, cancer, and other causes. Lancet.

[13] Rudnick MR, Goldfarb S, Wexler L (1995). Nephrotoxicity of ionic and nonionic contrast media in 1196 patients: a randomized trial. The Iohexol Cooperative Study. Kidney Int.

[14] Solomon R, Werner C, Mann D, D'Elia J, Silva P (1994). Effects of saline, mannitol, and furosemide on acute decreases in renal function induced by radiocontrast agents. N Engl J Med.

